# Duverney’s Fracture Fixed by Skiver Screw in a Skeletally Immature Patient: A Case Report

**DOI:** 10.7759/cureus.66939

**Published:** 2024-08-15

**Authors:** Jomon De Joseph, Ganesh Krishnan KR, Venkataram V

**Affiliations:** 1 Orthopedics, Mahatma Gandhi Medical College and Research Institute, Pondicherry, IND; 2 Orthopedics and Traumatology, Mahatma Gandhi Medical College and Research Institute, Pondicherry, IND

**Keywords:** screw fixation, pelvis acetabulum, pelvis fracture, fractures of the pelvis and acetabulum, pelvic fracture fixation

## Abstract

Duverney’s fracture, an eponym for isolated iliac wing fracture, is rather an uncommon fracture in the subset of pelvic ring fracture in the AO/OTA (Arbeitsgemeinschaft für Osteosynthesefragen/Orthopaedic Trauma Association) classification. Here, we discuss a more unique case with Duverney’s fracture since the patient is a 15-year-old school-going girl with unfused physis of the ilium. After other injuries were ruled out, she underwent open reduction and fixation with a screw by the Skiver method augmented with the plate. Postoperatively, she was mobilized, and follow-up at eight months showed radiologically complete union with good functional outcome.

## Introduction

Isolated iliac wing fracture makes up 2% of all pelvic fractures [1]. The fracture of the iliac wing is known as Duverney’s fracture, named after Joseph-Guichard Du Verney, who published his description of this particular fracture in the book "Traité des maladies des os" in 1751 [2]. Usually not seen as an isolated fracture, it is associated with acetabulum fracture or complication of pelvic surgery like iliac crest bone graft and total hip replacement [3,4]. It is also seen in the elderly with trivial falls [5]. Since the force required to create this injury is high velocity, it requires the need to rule out other concomitant injuries (intra-pelvic organ injuries [1] and abdominal wall injuries) [6] and complications associated with the fracture (major bleeding from venous plexus, arterial bleed [7], and Morel-Lavallee lesion [1]). The AO/OTA (Arbeitsgemeinschaft für Osteosynthesefragen/Orthopaedic Trauma Association) classification brings it under 61A2.1 in the pelvic ring category where the pelvic ring is not broken. Treatment options are nonoperative [6] and operative management [8]. For the fixation of this fracture, the Skiver screw technique has been described as a bone-air-bone method by the subiliacus approach [8]. Follow-up in the literature does not report any case of nonunion, malunion, infection, and myositis ossificans [6]. The case discussed here is unique in terms of isolated iliac wing fracture in a skeletally immature patient, which has not been described in the previous literature treated by operative intervention.

## Case presentation

A 15-year-old school-going girl was brought to the emergency department with an alleged history of road traffic accident. The initial assessment was done according to the Advanced Trauma Life Support (ATLS) protocol and the pelvic compression test was found to be positive on the right side. No neurological deficit was noted. History elaborated by the patient describes her being hit by a four-wheeler and having a direct impact on her right hip. Following this, she had pain in her right hip region on weight-bearing on her right lower limb. She was transported in a stretcher by an ambulance. A plain radiograph of the pelvis with both hips anteroposterior view was taken.

**Figure 1:**
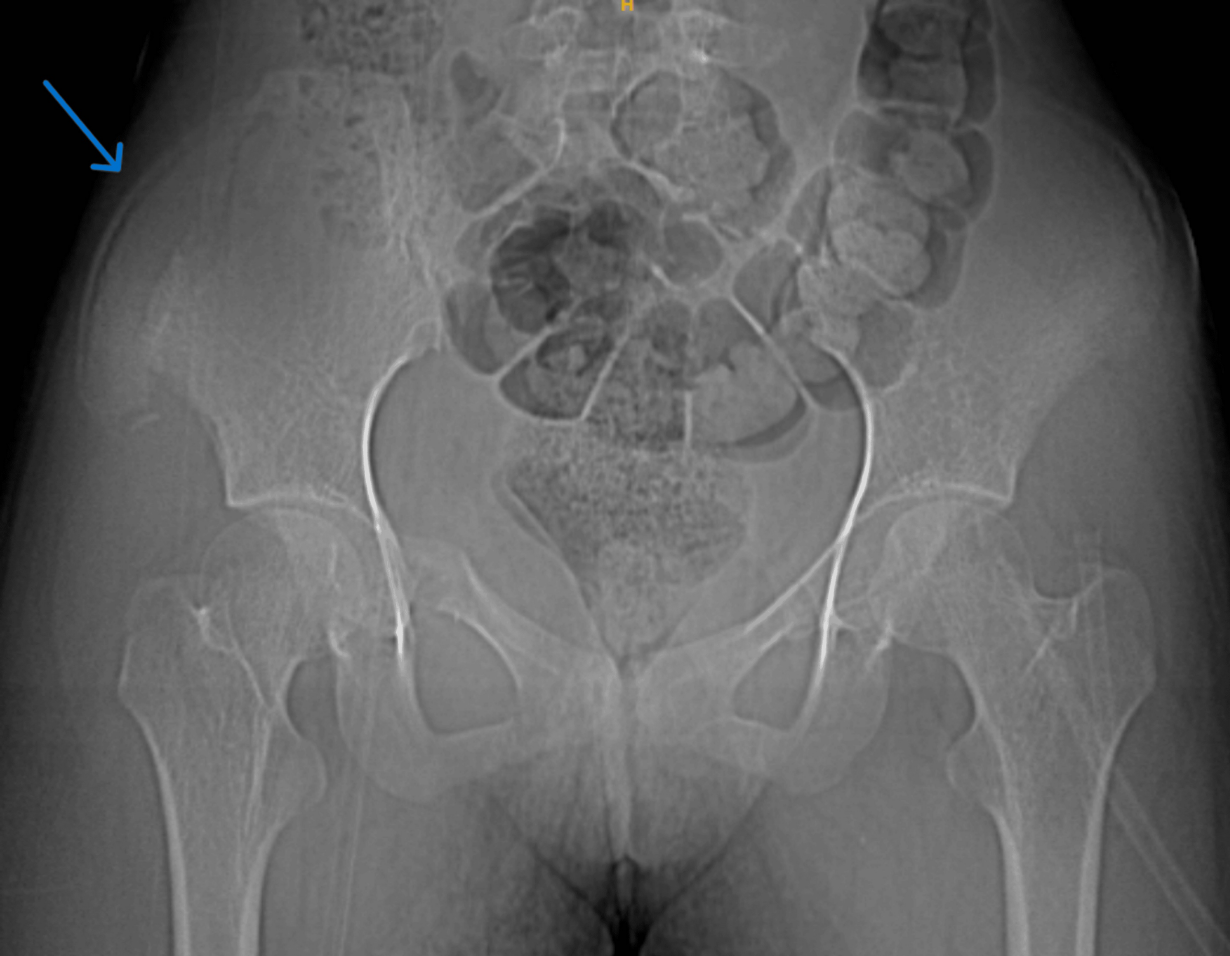
Plain anteroposterior radiograph of the pelvis with both hips. A plain X-ray showed an ilium fracture on the right side, marked by a blue arrow, and a superior and inferior pubic rami fracture on the right side.

X-ray showed a displaced right side iliac wing fracture with right side superior pubic rami fracture (Figure 1). For further evaluation, a plain CT of the pelvis was done.

**Figure 2:**
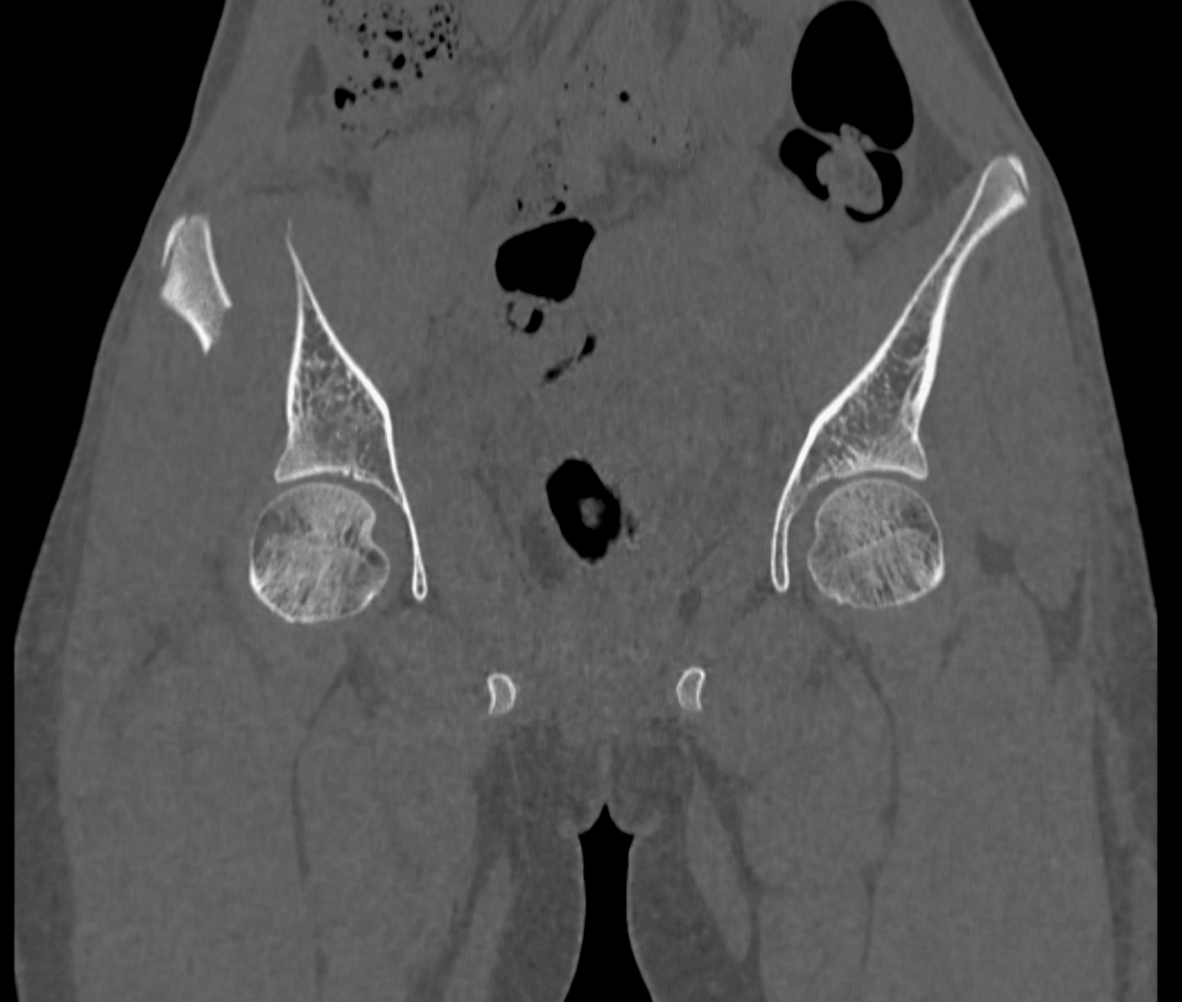
CT scan of pelvis anterior cuts. CT showed a displaced fracture of the right ilium in the anterior part of the right ilium.

**Figure 3:**
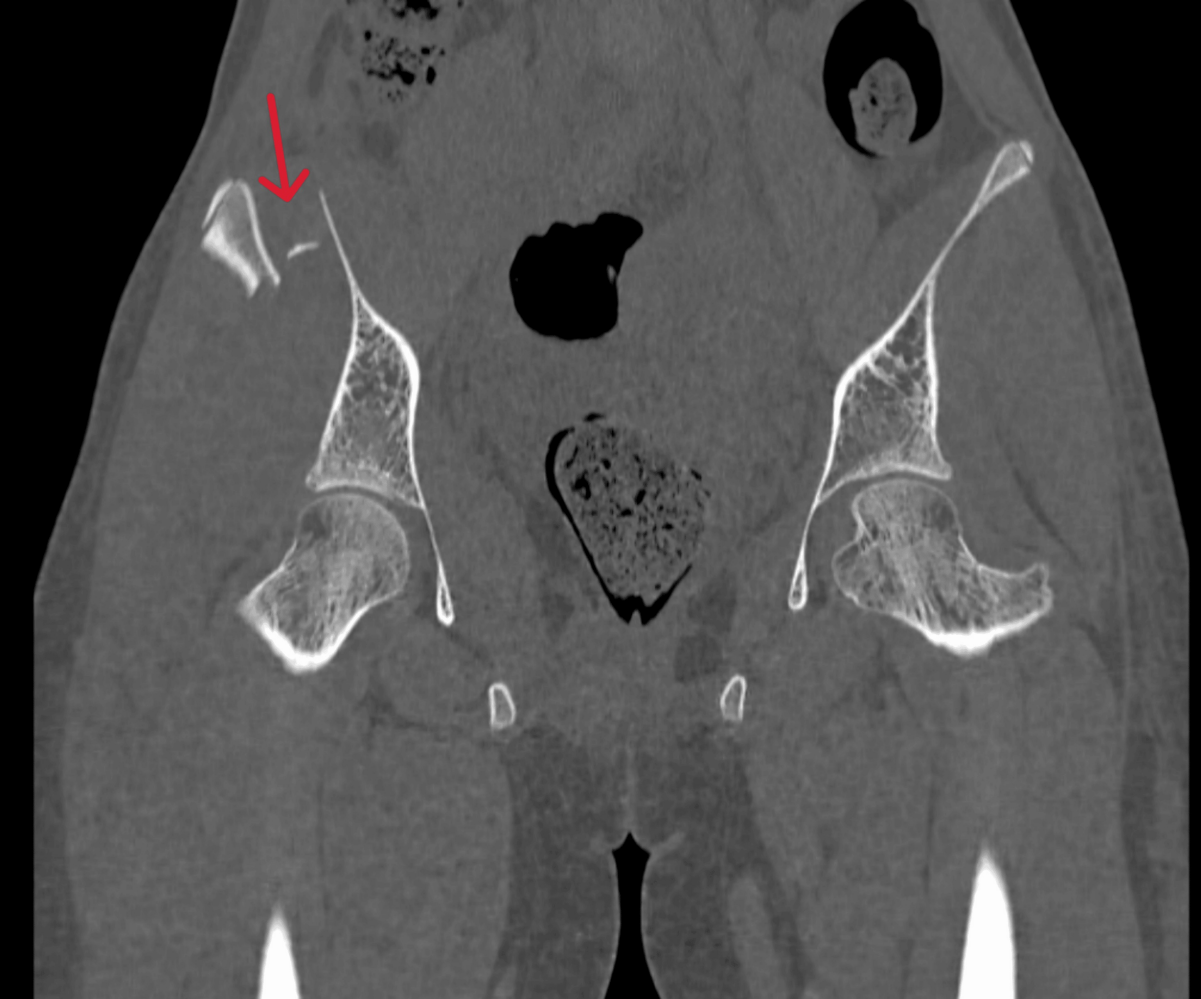
CT scan of pelvis posterior cuts. The coronal section of the posterior segment of the fracture shows comminution of the right ilium with a small fragment (red arrow).

CT scan was done (Figures 2, 3) to confirm the same and rule out other fractures of the pelvis and aid in the classification of the injury. The AO/OTA classification puts the above fracture under the category 61A2.1 group.

Since the patient is an active individual, we planned for operative management for early mobilization. She was planned for open reduction and internal fixation with a screw for the right ilium after anesthesia evaluation. Considering the three-dimensional anatomy of the ilium with the thickness of the bone being small, we planned for “the Skiver screw” technique. This technique, when applied perpendicular to the fracture line, gives compression by the lag screw principle. An additional plate was kept as a reserve due to the comminution noted in the posterior part of the fracture.

The patient was kept in the supine position under neuraxial anesthesia, and a skin incision along the iliac crest was followed by an intermuscular plane between the external oblique medially and tensor fascia lata with gluteus medius laterally. A periosteal elevator was used to make a subperiosteal plane from the iliac crest exposing the fracture. The fracture was reduced and provisionally held by Kirschner wires. The Skiver screw fixation for the anterior segment was done, followed by the posterior segment of the fracture, which was fixed by an eight-holed reconstruction plate considering the comminution.

One 4-mm cannulated cancellous screw was used for this technique. This technique decodes one of the many challenges in fixing the ilium considering the three-dimensional anatomy of the bone. The ilium is a curved bone with concavity on the medial aspect of the bone, and the minimal thickness of the bone prohibits a good length for the screw in a plate construct. Also known as the “in-out-in technique,” the screw starts from the iliac crest entering the iliac fossa and taking the distal purchase again in the ilium. This technique gets its name from the drill and the screw “skiving” into the iliac fossa after crossing the iliac crest. The screw position was confirmed under fluoroscopy with anteroposterior inlet and outlet views. The wound was closed in layers over the drain, which was removed in two days.

**Figure 4:**
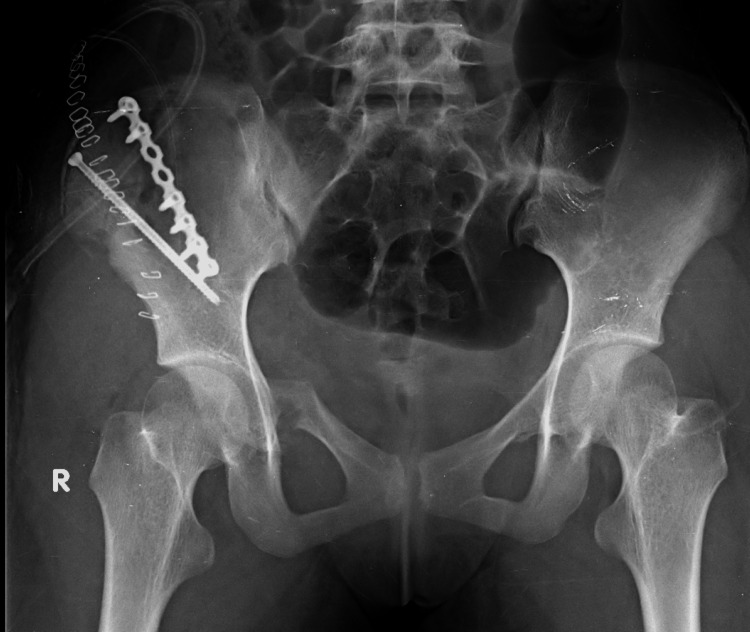
Postoperative anteroposterior radiograph of the pelvis. Immediate postoperative X-ray of the pelvis showed the Skiver screw in the anterior right ilium with the plate in the posterior right ilium.

**Figure 5:**
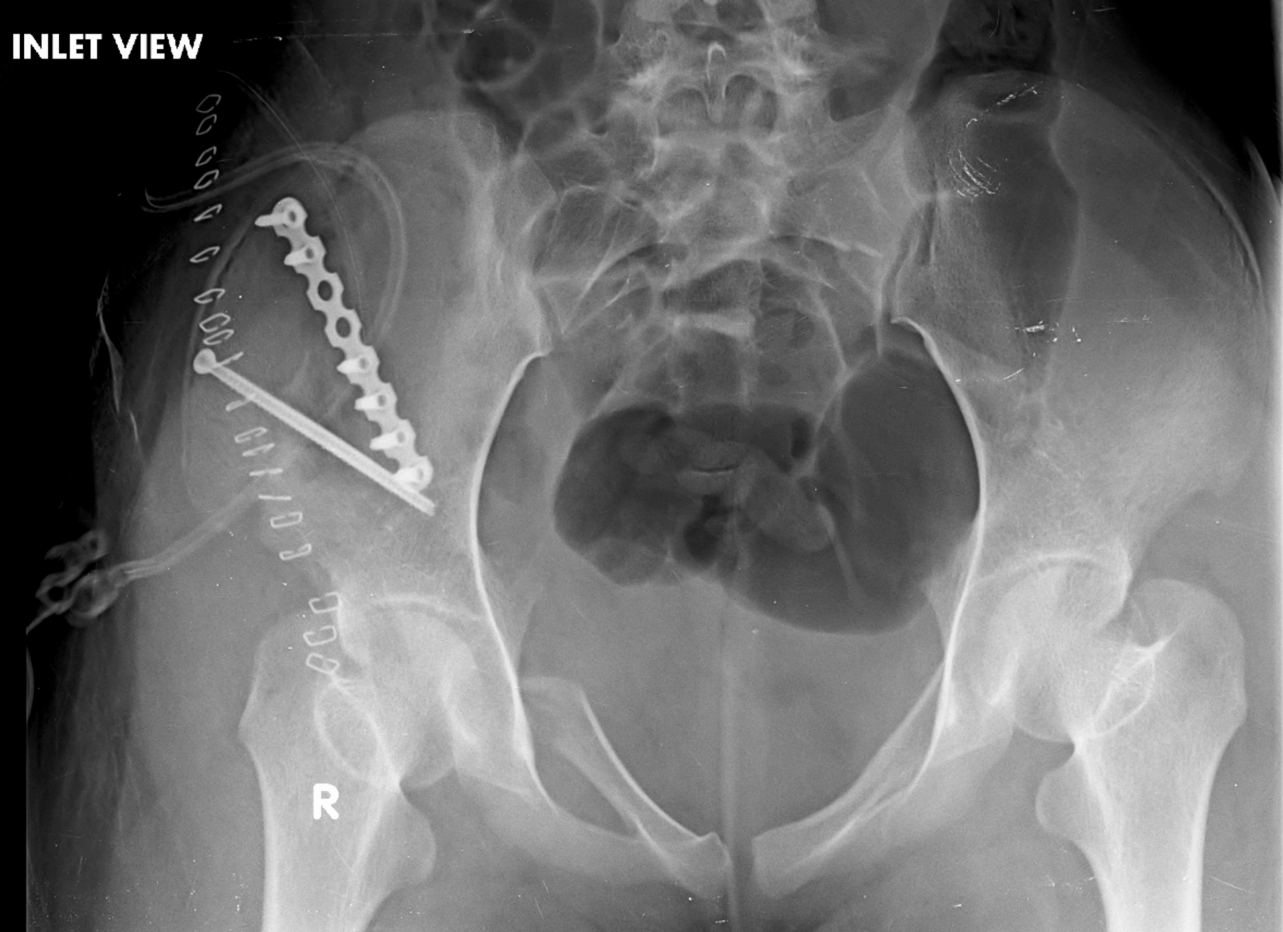
Postoperative inlet view of the pelvis. Inlet view showing fracture fixation and implant position.

**Figure 6:**
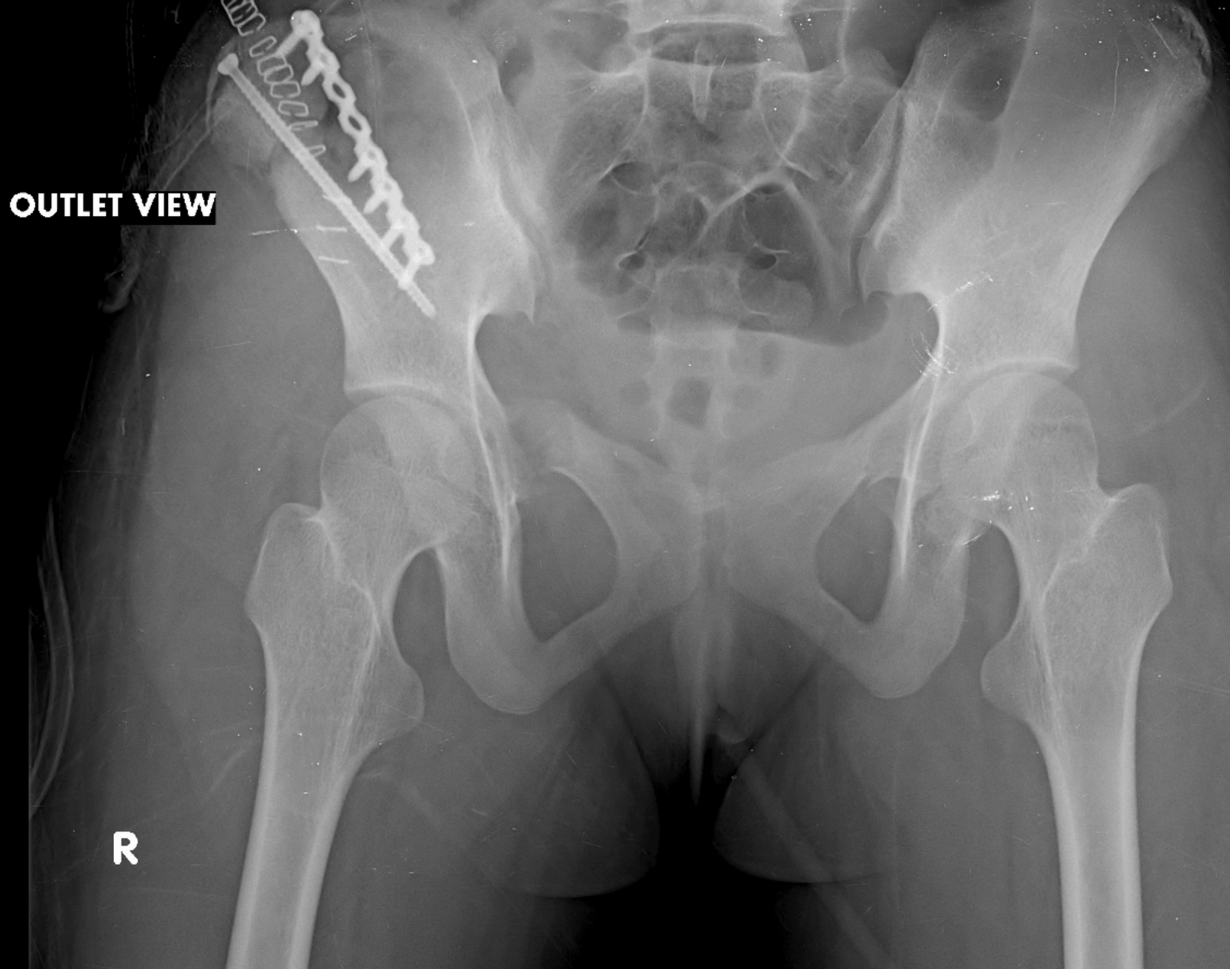
Postoperative outlet view of the pelvis. Outlet view showing fracture fixation and implant position.

**Figure 7:**
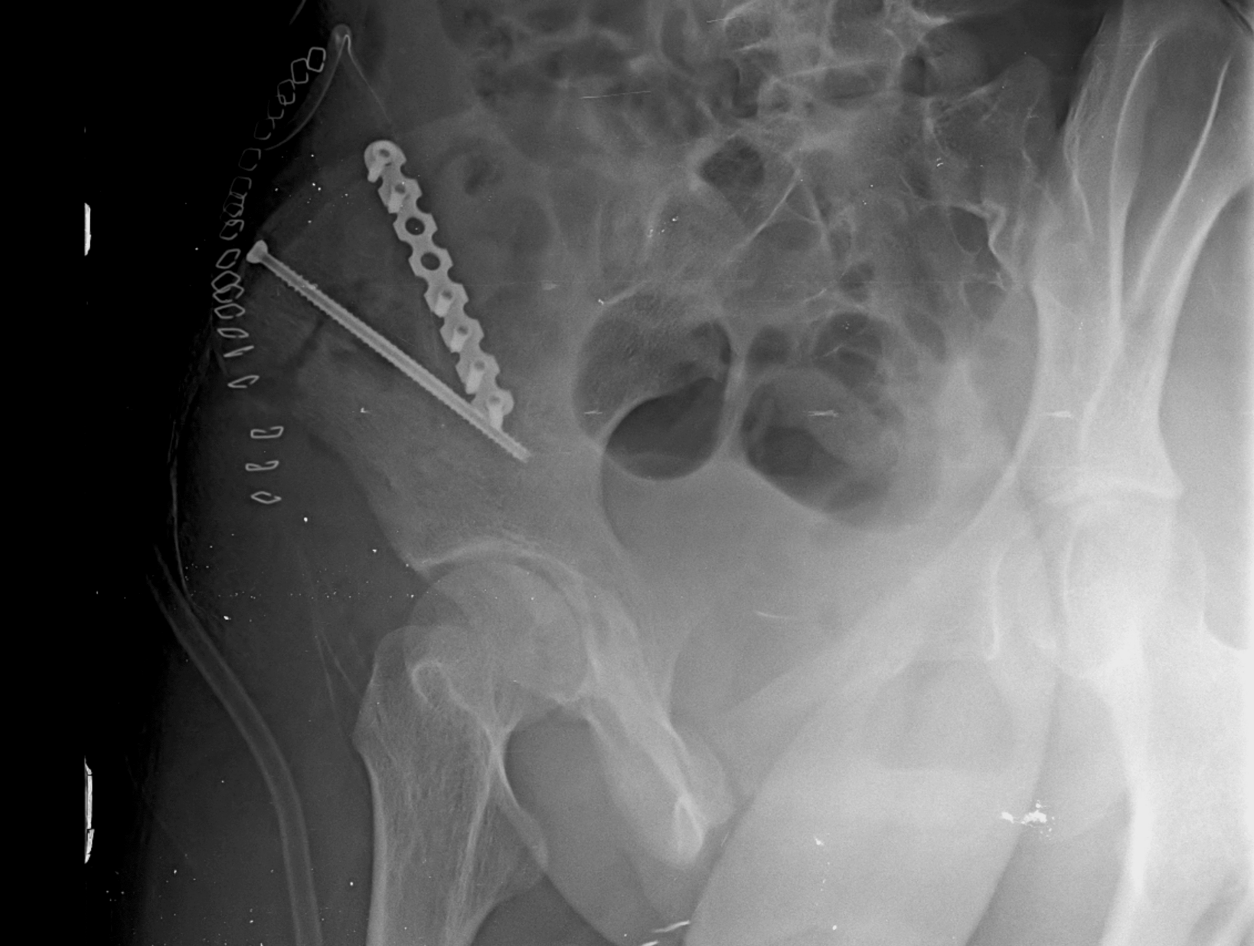
Postoperative Judet (right Iliac) view of the pelvis. The right iliac view (Judet view) shows good exposure of the right ilium with the Skiver screw and the plate.

Postoperative X-rays (Figure 4-7) were done to confirm implant position and she was mobilized non-weight bearing considering the presence of pubic rami fracture. Weight-bearing was gradually started from six weeks postoperatively, with partial weight-bearing and full weight-bearing from three months postoperatively.

**Figure 8:**
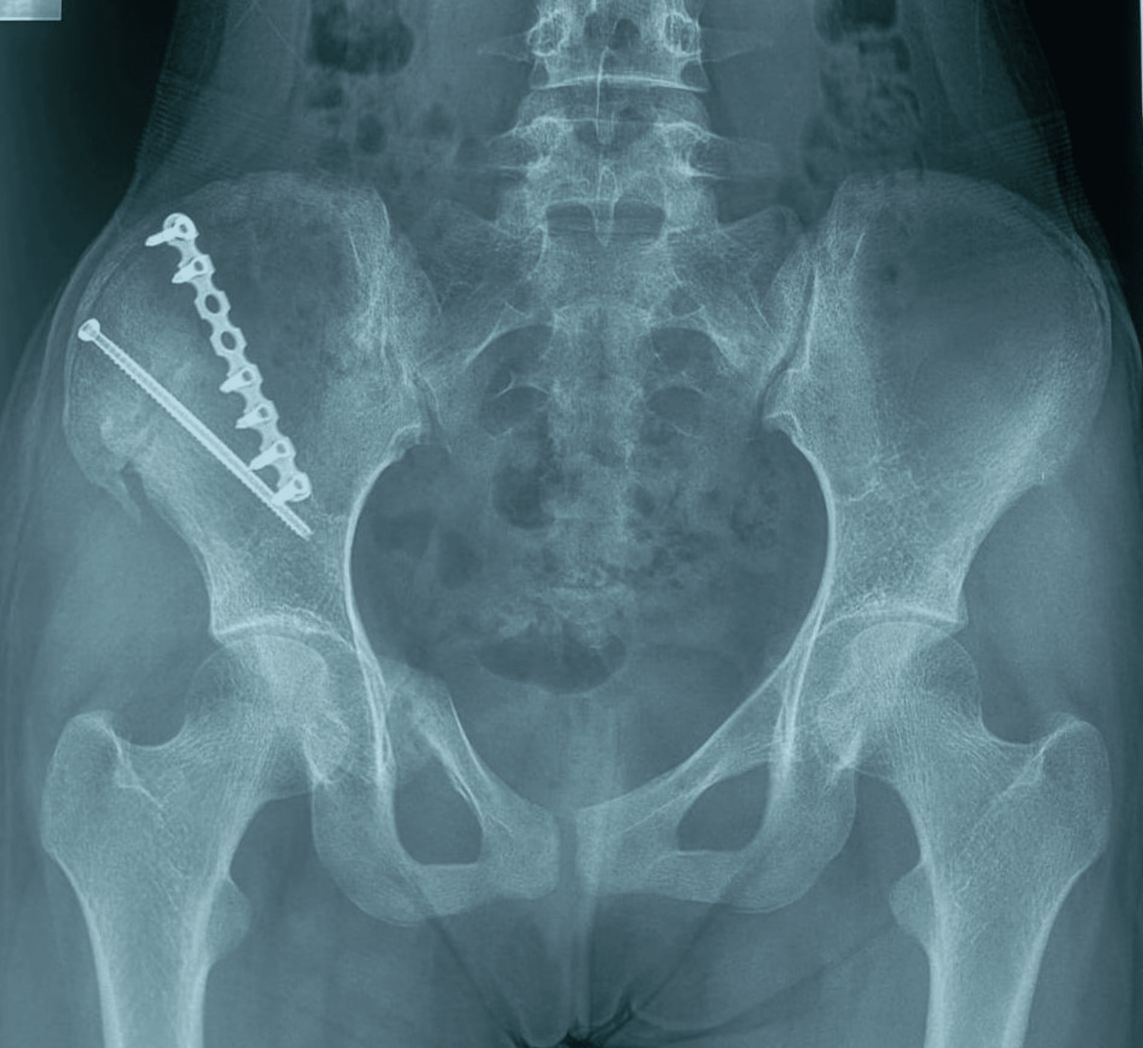
Six-month follow-up plain radiograph of the pelvis (anteroposterior view). Postoperative radiograph shows the fracture united well with the implant in a good position.

On six months follow-up, her radiograph showed complete fracture union (Figure 8). The patient is doing her day-to-day activities, including cross-leg sitting. She is a dancer and has started dancing from six months postoperatively.

## Discussion

The AO/OTA fracture and dislocation classification compendium puts this fracture as 61A2.1 [9]. This case report presents a rare instance of Duverney’s fracture, an isolated iliac wing fracture, in a skeletally immature patient, successfully treated with the Skiver screw technique. Duverney's fractures are uncommon, constituting only about 2% of pelvic fractures [1], and are typically associated with high-energy trauma [1]. In this case, a 15-year-old girl sustained an injury in a road traffic accident. The uniqueness of this case is the patient's age and skeletally immature status combined with operative intervention and good functional outcome.

Literature review

The literature presents limited case reports about traumatic isolated iliac wing fractures, especially non-athletic types of injuries. There is a special consideration for the iliac crest for adolescent patients. The cartilaginous iliac crest apophysis is weaker than the musculotendinous attachment. The avulsion fracture of the iliac crest seen in this case is explained by forceful contraction of the muscle insertion, namely, sartorius and tensor fascia lata [10].

The pelvic ring integrity is not broken in this type of fracture. Thereby making it a stable fracture [11]. Also, according to the AO classification, this belongs to stable pelvic ring fracture, which is usually treated non-operatively. However, avulsion-type fractures have been mentioned to benefit from operative treatment. The relative indication mentioned in the literature is to reduce pain due to fracture mobility and improve functional outcomes. Cosmesis is another factor mentioned [10].

Anatomical reduction of the fracture with rigid fixation permits mobilization since it prevents pain due to fracture, which can be present even during in-bed mobilization [10,11]. There are limited options for fracture fixation with non-operative management like spica cast giving concerns about complications of immobilization like deep vein thrombosis [11]. Recommended choice of the construct for fixation is a compression screw [10].

Isolated compression screw without additional construct is described as sufficient for stable fixation [10]. However, the fracture pattern described in our case warrants plate augmentation in the posterior segment due to the comminution present, which is unique in this case. This adds a note to the type of fracture in this case report since existing literature describes avulsion fractures only [10].

Compression screw by Skiver screw method and plate augmentation aligns with the principles of achieving anatomical reduction and stable fixation to facilitate early mobilization and rehabilitation. Non-union, malunion, infection, and myositis ossificans are the potential complications of pelvic fractures, though not commonly reported with isolated iliac wing fractures [6].

Clinical significance and challenges

The management of pelvic fractures in children poses unique challenges due to the presence of unfused growth plates and the potential question for long-term sequelae affecting growth and development. In this patient, the initial presentation included pain and the inability to bear weight on the affected side, with radiographic and CT imaging confirming the iliac wing fracture and a concomitant superior pubic rami fracture. The choice of the Skiver screw technique was particularly suitable given the three-dimensional anatomy of the ilium and the need for stable fixation.

The Skiver screw technique

The word ''Skiver'' in this technique indicates how the screw behaves in its trajectory while passing through the ilium. The Skiver screw technique, or the "in-out-in" method, is advantageous for its ability to provide stable fixation in the curved and thin bone of the iliac wing. This technique has been described for inferior glenoid and scapular neck fractures [8]. We have used the same technique for this case. The screw enters the iliac crest initially then traverses the iliac fossa and re-enters the ilium. This provides absolute stability to the fracture by the lag screw principle [8]. The use of a cannulated cancellous screw ensures better control of trajectory and compression at the fracture site.

Postoperative management and outcomes

Postoperative care involved initial non-weight-bearing mobilization to accommodate the additional pubic rami fracture, transitioning to weight-bearing at six weeks post surgery. The successful union of the fracture was confirmed radiographically at eight months, with the patient returning to normal activities, including sports. This outcome highlights the effectiveness of the Skiver screw technique in providing stable fixation and promoting fracture healing in a pediatric patient.

This case contributes to the existing body of knowledge by documenting a successful surgical intervention by the Skiver screw method for Duverney's fracture in a pediatric patient. It underscores the importance of tailored surgical techniques to address the anatomical and developmental considerations in children. The positive functional outcome in this case supports the Skiver screw technique as a viable option for similar fractures in the pediatric population.

## Conclusions

The treatment of Duverney’s fracture in a skeletally immature patient with the Skiver screw technique demonstrates a successful outcome, with complete fracture union and return to pre-injury activity levels. This case highlights the need for careful preoperative planning and selection of appropriate fixation techniques to address the unique challenges presented by pelvic fractures, especially in a skeletally immature patient. Further studies and case reports will be valuable in consolidating the evidence for optimal management strategies in this rare injury.
